# Current Trends in the Management of Port-Site Infections: A Case Series and a Review of Published Work

**DOI:** 10.7759/cureus.40936

**Published:** 2023-06-25

**Authors:** Ramkaran Chaudhary, Vibhor Tak, Akshat Dutt, Mahaveer S Rodha, Satya Prakash Meena, Mayank Badkur, Sarika P Kobade, Mahendra Lodha, Naveen Sharma, Vijaya L Nag, Ashok Puranik

**Affiliations:** 1 General Surgery, All India Institute of Medical Sciences, Jodhpur, Jodhpur, IND; 2 Microbiology, All India Institute of Medical Sciences, Jodhpur, Jodhpur, IND; 3 Surgery, Jawaharlal Institute of Postgraduate Medical Education and Research, Puducherry, IND; 4 General Surgery, All India Institute of Medical Sciences, Guwahati, Guwahati, IND

**Keywords:** minimal access surgery, atypical mycobacteria, laparoscopic surgery, non-tuberculous mycobacterium, port-site infections

## Abstract

Introduction

Laparoscopic techniques have become standard for many surgeries, offering benefits such as quicker recovery and less pain. However, port-site infections (PSIs) can occur and pose challenges. PSIs can be early (within seven days) or delayed (after three to four weeks), with delayed PSIs often caused by non-tuberculous mycobacteria (NTMs). NTMs are difficult to treat and do not respond well to antibiotics, leading to prolonged and recurrent infections. Guidelines for PSI management are limited. This summary highlights a case series of 10 patients with PSIs, discussing their treatment experience and presenting a treatment algorithm used at our institute.

Methods

This is a retrospective study (2015-2020) on chronic port-site infections (PSIs) in laparoscopic surgeries. Data were collected on patient demographics, surgery type, prior treatment, and management at the institute.

Results

The study analyzed 10 patients with chronic PSIs following laparoscopic surgery between 2015 and 2020. Laparoscopic cholecystectomy was the most frequent index surgery. Three patients had a history of treatment with varying durations of anti-tubercular therapy, one of whom had completed anti-tubercular treatment prior to presentation. Complete surgical excision with histopathological examination and fungal, bacterial and mycobacterial cultures were performed. Seven of the 10 patients were treated with oral ciprofloxacin and clarithromycin combination therapy for three months, two were treated with culture-based antibiotics and one was treated with anti-tubercular therapy. All patients improved on treatment. The mean follow-up period was 52 ± 9.65 months, with no relapses being reported.

Conclusion

Port-site infections (PSIs) are troublesome complications of laparoscopic surgery that can erode the benefits of the procedure. Delayed PSIs caused by drug-resistant mycobacteria are difficult to treat. Improved sterilization methods and thorough microbiological work-up are crucial. Radical excision and prolonged oral antibiotics are effective treatments. Clinicians should avoid empirical antibiotic therapy to prevent antimicrobial resistance.

## Introduction

Since their inception, laparoscopic techniques have become increasingly popular and are now the standard of care for many surgeries [1,2]. Laparoscopic techniques have been deployed in a wide array of surgeries, ranging from relatively simple procedures such as cholecystectomies to more complicated ones like radical prostatectomy, with encouraging outcomes [3,4]. In spite of their numerous advantages such as early recovery, better cosmesis, and lower post-operative pain, they are not without complications. Port-site infections (PSIs) are a set of chronic, nagging, treatment-refractory complications that are unique to laparoscopic procedures. They result in significant morbidity and erode the benefits of laparoscopic procedures. PSIs may be classified as early, when presenting within seven days of surgery, or delayed when presenting after three to four weeks of surgery [5]. Early PSIs are mostly caused by skin commensals and respond to treatment with empirical antibiotics. However, delayed PSIs are elusive entities that evade diagnosis by routine microscopic and culture techniques [6]. Non-tuberculous mycobacteria (NTMs), otherwise called atypical mycobacteria, or mycobacteria other than tuberculosis (MOTT) are commonly implicated in delayed PSIs [6]. This occurs primarily due to two reasons. Firstly, NTMs are fastidious organisms that evade sterilization by routine techniques. Secondly, laparoscopic instruments have a layer of heat insulation that further decreases the efficacy of routine heat-based sterilization. Moreover, NTMs do not respond to treatment with several antibiotics and are known to cause frequent relapses [7]. Thus, the treatment of most cases of delayed PSIs follows a tortuous course and leads to a protracted morbid state [6,8]. There is a paucity of established guidelines regarding the management of PSIs. We report our experience in managing PSIs through a series of 10 patients treated at our institute. We also include a treatment algorithm that we followed at our institute to manage these PSIs.

## Materials and methods

This is a retrospective review of the medical records of 10 patients who presented with chronic port-site infections at All India Institute of Medical Sciences, Jodhpur between 2015 and 2020. The study was initiated after obtaining approval from the institutional ethics committee. The medical records were reviewed for patient characteristics and clinical details. Data obtained were anonymised and all patient identifiers were removed. Data including index surgery, treatment received prior to presentation, imaging findings, laboratory reports, and antibiotic therapy offered at our institute were collected. Microbiological and pathological records were retrieved for all the patients from the computerised hospital information system. Microbiological records were screened for evidence of acid-fast bacilli on microscopy, and the presence of non-tuberculous mycobacteria (NTM) on microbiological culture. Pathological records were reviewed for the presence of necrotising granuloma on histopathological examination.

**Figure 1:**
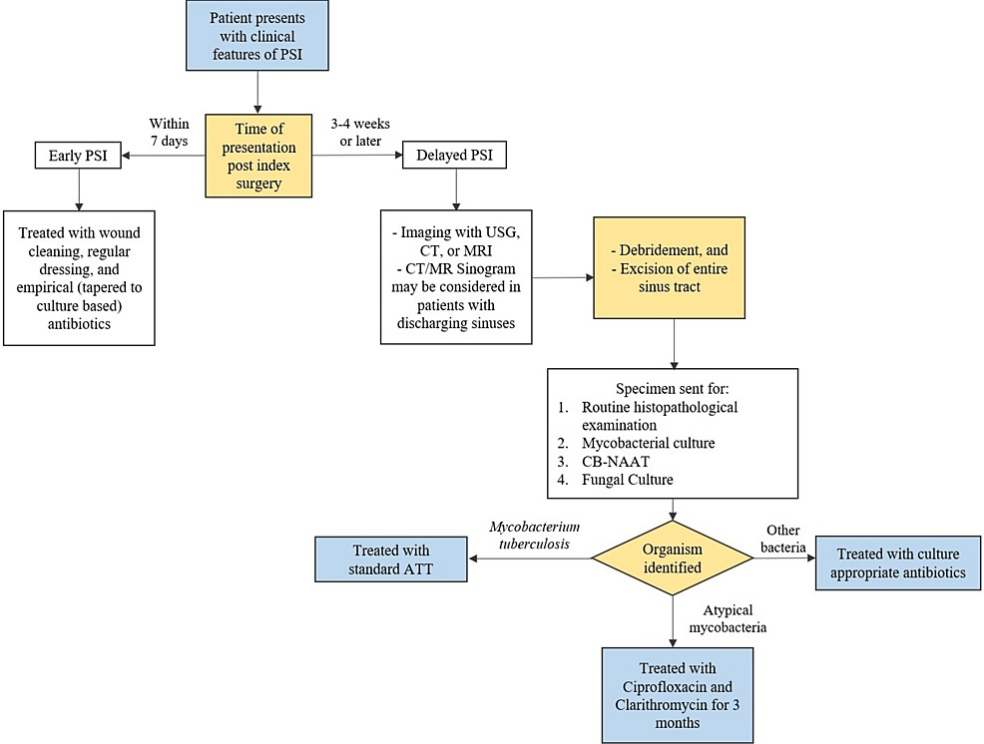
Departmental treatment algorithm for port-site infections following laparoscopic surgery PSI: Port-site infection; USG: Ultrasonography; CT: Computerised tomography; MRI: Magnetic resonance imaging; CB-NAAT: Cartridge-based nucleic acid amplification test; ATT: Antitubercular therapy

Chronic port-site infections were identified clinically by the presence of non-healing wounds, persisting discharge, and/or wound breakdown. Patients in whom a minimal access procedure was attempted, even if it was abandoned for an open approach later, were included in the study. Once the patients were diagnosed with a chronic port-site infection, they were managed according to a predetermined departmental protocol as detailed in Figure 1. The patients underwent screening ultrasonography (USG) to delineate the extent of the collection of the sinus tracts, while computerised tomography (CT) and magnetic resonance imaging (MRI) were reserved for diagnostic ambiguity. The patients underwent complete surgical excision of the wound with a thorough dissection of the entire sinus tract. The specimens were sent for routine histopathological examination, fungal, bacterial, and mycobacterial cultures. The surgical wounds after debridement were left to heal with secondary intention and negative pressure wound therapy was administered in suitable cases. The patients were not started on empirical antibiotic therapy before the cultures and histopathological examination were reported. Data were analysed using Statistical Program for Social Sciences (SPSS version 26.0 for Windows; IBM Corp., Armonk, NY).

## Results

**Figure 2:**
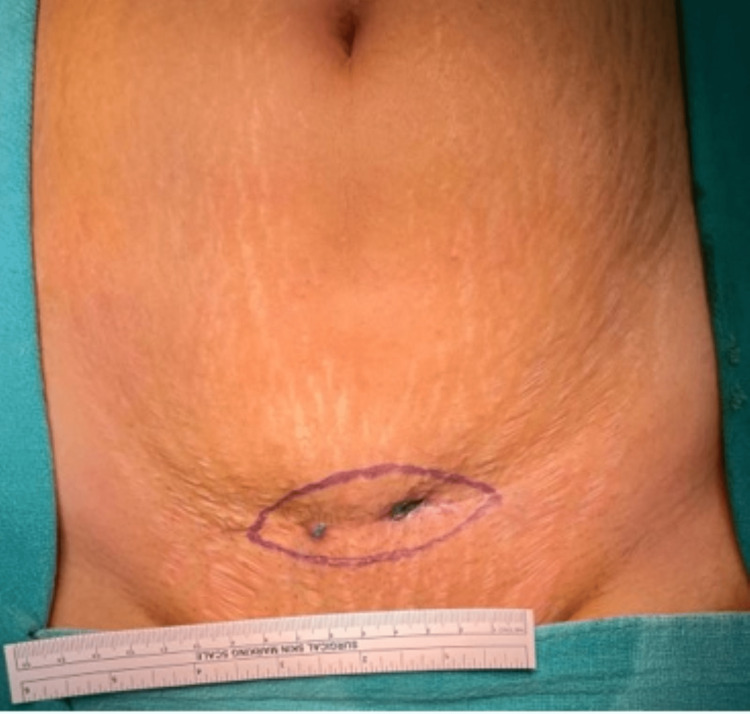
Image showing persistent discharging sinuses that developed in a patient with delayed port-site infection

**Figure 3:**
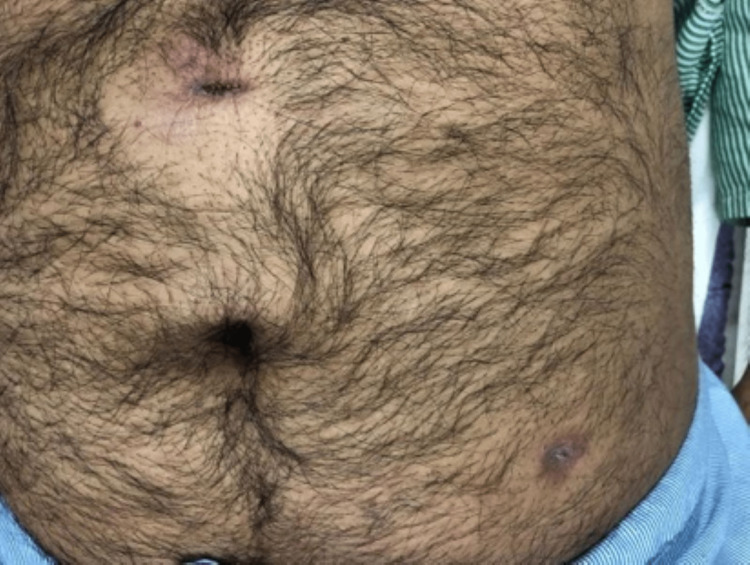
Image showing infected epigastric and left lumbar ports in a patient who had undergone laparoscopic cholecystectomy

A total of 10 patients presented to our institute with chronic PSIs after complete laparoscopic surgeries between 2015 and 2020. None of these patients had undergone their index surgeries at our institute. There were seven females and three males included in the study. The mean age of the patients was 36 ± 15 years, ranging from 17 to 65 years. The most frequent index surgery was laparoscopic cholecystectomy (30%), followed by laparoscopic appendicectomy (20%) (Table 1). Three patients had received varying durations of anti-tubercular therapy, with one patient having completed treatment before presentation. The details of presentations and management at our centre have been summarised in Table 1. Figures 2-3 depict how a chronic port-site infection appeared in two of our patients.

**Table 1:** Demographic and treatment details of the patients of port-site infections who presented to our hospital Abbreviations used: ATT: Anti-tubercular therapy; HPE: histopathological examination; AFB: acid-fast bacillus; MRSA: methicillin-resistant Staphylococcus aureus; CONS: coagulase-negative staphylococci

Serial	Age/ Sex	Previous Surgery	ATT (Duration)	Imaging	Microscopy	Culture	HPE	Antibiotic therapy
1.	65/M	Laparoscopic appendicectomy	No	No	AFB	NTM	Necrotising granulomatous inflammation	Ciprofloxacin PO 3 months Clarithromycin PO 3 months
2.	40/F	PCNL	Yes (6 months)	USG: Sinus tract in left lumbar lesion	AFB	MRSA	Chronic inflammation	Linezolid PO 5 days Amoxycillin/Clavulanate PO 5 days
3.	29/F	Laparoscopic Ovarian Cystectomy	No	USG: Cellulitis of LIF and iliac wall	Negative	Negative	Chronic inflammation	Ciprofloxacin PO 3 months Clarithromycin PO 3 months
4.	53/F	Laparoscopic cholecystectomy	Yes (4 days)	MRI: Fistulous tract upto subhepatic and posterior pararenal space	Negative	Negative	Chronic inflammation	Ciprofloxacin PO 3 months Clarithromycin PO 3 months
5.	18/F	Laparoscopic appendicectomy	No	CT: Soft tissue thickening in skin and subcutaneous tissue in epigastrium	Negative	MRSA	Chronic inflammation	Linezolid PO 5 days Amoxycillin/Clavulanate PO 5 days
6.	45/F	Laparoscopic cholecystectomy	No	USG: Linear hypoechoic tract up to anterior rectus sheath in infra-umbilical region	Negative	Negative	Chronic inflammation	Ciprofloxacin PO 3 months Clarithromycin PO 3 months
7.	31/F	Laparoscopic oophorectomy	No	CT: patchy fibrosis of anterior abdominal wall at the previous site of laparoscopy ports	Negative	CONS	Necrotizing granulomatous inflammation	Ciprofloxacin PO 3 months Clarithromycin PO 3 months
8.	27/M	Laparoscopic hernia repair (TEP)	Yes (2 months)	No	Negative	Negative	Chronic inflammation	Continuation of ATT for 4 months
9.	38/F	Laparoscopic cholecystectomy	No	USG: hypoechoic longitudinal collection is in the subcutaneous plane; CT: Linear soft tissue thickening tract abutting the muscle layer	Negative	CONS	Necrotizing granulomatous inflammation	Ciprofloxacin PO 3 months Clarithromycin PO 3 months
10.	17/M	Laparoscopic converted to open appendicectomy	No	USG: Two linear tracts from RIF to surgical scar	Negative	Negative	Necrotizing granulomatous inflammation	Ciprofloxacin PO 3 months Clarithromycin PO 3 months

Four of the 10 patients demonstrated evidence of bacterial infection. Three patients had necrotising granulomas upon histopathological examination, one of whom had acid-fast bacilli upon microscopic examination, and grew NTMs on mycobacterial culture. There was one more patient who had acid-fast bacilli upon microscopic examination but did not demonstrate further evidence of non-tuberculous mycobacteria. The cultures of two patients yielded MRSA. The imaging, microbiological, and histopathological work-up of the patients have been detailed in Table 1.

Three patients had already received standard anti-tubercular treatment (ATT) in the past, one of whom had gone on to complete their course of treatment before presentation. One other patient had received ATT for two months, and he was treated with continuing ATT for four more months. One patient had been placed on ATT without any evidence of mycobacterial infection, and for her ATT was stopped.

Seven of the 10 patients were treated with oral ciprofloxacin and clarithromycin combination therapy for three months. One patient who had completed their ATT was treated with oral linezolid and amoxicillin-clavulanate combination therapy according to the culture reports. One patient who tested positive for MRSA on aerobic culture received treatment with oral linezolid and amoxicillin-clavulanate. All patients improved on receiving antibiotic therapy and were asymptomatic on follow-up. The patients received a mean follow-up of 52 ± 9.65 months. There were no treatment relapses reported.

## Discussion

Albeit relatively uncommon, port-site infections are a frustrating complication of laparoscopic surgery. Most of the laparoscopic procedures performed fall into the clean or the clean-contaminated categories of surgical wounds as described by the CDC [9,10]. Despite the relatively clean nature of these surgical wounds, the reported incidence of port-site infections in the literature ranges between 1.4 and 6.3% [9]. The source of infection may be endogenous or exogenous. Endogenous sources of infection may be reduced by adequate preoperative bowel preparation, and by containing spillage during surgery. The latter may be achieved by minimizing hollow viscus injuries and using endobags for specimen retrieval [11,12]. On the other hand, exogenous source reduction may be effectuated by sedulous sterilization. Recent evidence suggests that a breach in the sterilization protocol is the most common cause of PSIs due to non-tuberculous mycobacteria [5]. NTMs occur widely in nature, including in soil and running water, due to which they easily contaminate hospital instruments. Infections with NTMs have been reported primarily after laparoscopic surgeries [13]. This may be explained by two major factors. Firstly, laparoscopic instruments have a layer of insulation that limits the use of autoclaving in their sterilization, resulting in incomplete elimination of NTM endospores. Secondly, laparoscopic instruments have multiple joints and moving parts where biological soil, charred tissue, and grime can accumulate. These require meticulous cleaning of the instruments before they can be sent for sterilization. Inadequacy in the cleaning process often results in the deposition of endospores in the instruments, which can be transferred to the patients during surgery.

Many centres in India immerse laparoscopic instruments in 2-2.5% glutaraldehyde solution for 20 minutes between surgeries [5]. At the aforementioned concentration and contact time, glutaraldehyde acts as a high-level disinfectant, but not a sterilant, thereby allowing the bacterial endospores to survive [14]. Current guidelines suggest that the chemical can only be used for a maximum of 100 cycles over 14 days (2.5% glutaraldehyde) or 28 days (3.4% glutaraldehyde) [5]. In practice, however, no count of the number of cycles is kept and glutaraldehyde often does not have the requisite potency for adequate sterilization. Moreover, it is not an uncommon practice to rinse off the glutaraldehyde with tap water, thereby reintroducing NTM endospores into the instrument, which are then deposited in the patients’ subcutaneous tissue. In order to achieve effective sterilization, laparoscopic instruments must be completely dismantled and then washed thoroughly to ensure the removal of biological soil [15]. If glutaraldehyde is used for sterilization, it must be of adequate strength (3.4%) and must have a prolonged contact time (8-12 hours) for sporicidal activity [5]. Finally, only autoclaved or sterile water should be used to rinse the laparoscopic instruments after exposure to glutaraldehyde. It is prudent to limit the widespread use of glutaraldehyde in the sterilization of laparoscopic instruments as it is associated with numerous pitfalls. Plasma sterilization systems such as STERRAD offer a cheap and effective alternative for low-temperature sterilization [5,16]. Other techniques such as ethylene oxide gas sterilization and formalin gas chambers may also be used with varying efficacy [5]. The most effective technique for the prevention of PSIs would be the usage of disposable laparoscopic instruments. However, the steep costs involved therein limit its utility in resource-constrained settings.

Once the NTM endospores have been inoculated into the patient, they gradually germinate over three to four weeks. This explains why these entities frequently present as delayed port-site infections. NTMs have an affinity for the dermis and subcutaneous tissue, whereas they are destroyed by the protective factors within the peritoneum [5]. There is little evidence of NTMs causing disseminated disease in an immunocompetent host [5,17]. The clinical presentation is varied, however, patients rarely present with systemic symptoms such as fever. Findings from recent studies pertaining to the index surgery, patient demographics, and clinical manifestations have been summarized in Table 2. Chaudhuri et al. described various stages of delayed PSIs, which have been summarized in Table 3 [5].

**Table 2:** A summary of recent studies reporting the patient demography, time to presentation, and clinical manifestations of port-site infections TTP: Time taken to present

Serial	Reference, Country; n	Surgery (n)	Demographics	Clinical Manifestation
1.	Ghosh et al. [6], 2017, India; n=32	Cholecystectomy (19); herniorrhaphy (1); orchiopexy (1); appendectomy (1); hemicolectomy (1); bile duct repair (1); diagnostic (1)	Male (12, 37%); Age (median 39, range 8-67 years)	TTP: 4-5 weeks induration, swelling serosanguinous discharge, sinus formation; no fever
2.	Ramesh et al. [14], 2003, India, n=8	Adhesiolysis (2); cholecystectomy (1); endometriosis (1); hernia repair (1); hysterectomy (2); oophorectomy (1)	Male (1, 12.5%); Age (median 28.5 years, range 25-67 years)	TTP: N/A; non-healing port-site wounds, where wounds had not healed even at more than 4 weeks of surgery.
3.	Shah et al. [18], 2009, n=7	Hernia repair (6); bowel resection (1)	NA	TTP: 10 days to 3 weeks; delayed onset wound infection, appearance of minimal erythema, oedema, wound breakdown, suppuration, and discharging sinuses. No fever or systemic symptoms
4.	Bhattacharjee et al. [19], 2015 India; n=55	Cholecystectomy (55)	Male (14; 25%); Age (median 35, range 15-75)	TTP: 4-6 weeks; tender induration, seropurulent discharge
5.	Chaudhuri et al. [5], 2010, India; n=19	Cholecystectomy (19)	Male (3; 16%); Age (median 43, range NA)	TTP: 3-4 weeks; erythematous swelling, tender; ultimate caseation and discharge; mo fever
6.	Duarte et al. [20]., 2009, Brazil; n=197	Cholecystectomy (71); diagnostic (10); appendicectomy (9); oophorectomy (4); herniorrhaphy (3); tubal ligation (3); other (25)	Male (105; 27%); Age (median 44, range 14-89 years)	TTP: 2-187 days; seropurulent discharge (61%); erythema (23%); nodule (23%), abscess (19.8%); fistula (6.3%); ulcer (3.9%); fever (11.1%)
7.	Vijayaraghavan et al. [21]., 2005, India; n=156	Laparoscopy (156)	NA	TTP: N/A, nodule formation, erythema fever
8.	Krishnappa and Samarasam [22] 2017 India; n=24	Cholecystectomy (10); herniorrhaphy (3); not specified (11)	Male (8; 33%); Age (mean 42 years, range NA)	TTP: N/A, erythematous swelling, no systemic signs
9.	Lahiri et al. [17], 2009, India; n=5	Tubectomy (3); herniorrhaphy (2)	Female (3; 60%)–underwent tubectomy Male (2; 40%) – underwent herniorrhaphy Age NA	TTP: under 4 weeks nodular swelling, discharging sinus
10.	Muthusami et al., 2004 [13], India; n=5	Cholecystectomy (4); appendectomy (1)	Male (2; 40%); Age (median 43; range 30-48 years)	TTP: N/A; discharging sinuses; unresponsive to empirical antibiotics
11.	Samaranayake and Dassanayake [23], 2018, Sri Lanka; n=3	Adhesiolysis LRT herniorrhaphy	Female (2; 67%); Male (1; 33%); Age (range 36-46 years)	TTP: 3-4 weeks; port-site discharge, swelling, erythema, sinus tract formation; No fever
12.	Baruque Villar et al. [24], 2015, Brazil; n=60	Cholecystectomy (55)	Male (15; 25%); Age (mean 40, range 20-82 years)	TTP: median 30 days (range: 7–150); discharge (80%); local pain (72%); nodules (51%); fever (18%); abscess (38%); fistula (15%)
13.	Wright et al. [25], 2014, Australia; n=18	Gastric banding (18)	Female (15; 83%); Male (3; 17%); Age (mean 45, range 29-64y)	TTP: 21 days-8 years pain, erythema band infection (fever, abdominal pain, and nausea)
14.	Current study (Chaudhary et al.)	Appendicectomy (3); cholecystectomy (3); hernia repair (1); oophorectomy (1); ovarian cystectomy (1); PCNL (1)	Male (3; 30%); Age (mean 36, range 17-65 years)	TTP: 6 weeks to 12 years; pain, tenderness, erythema, persistent discharging sinus

**Table 3:** Clinical staging of delayed port-site infections As described by Chaudhuri et al. [5].

Clinical Stage	Clinical Features
Stage 1	Tender nodules, in the vicinity of the port-site. Appears four weeks after surgery.
Stage 2	Nodules enlarge, become more tender and inflamed. A discharging sinus may form
Stage 3	Pain reduces after pus discharge. Overlying skin gets necrosed.
Stage 4	Chronic discharging sinus develops
Stage 5	Darkening of surrounding skin. Multiple nodules may appear.

**Figure 4:**
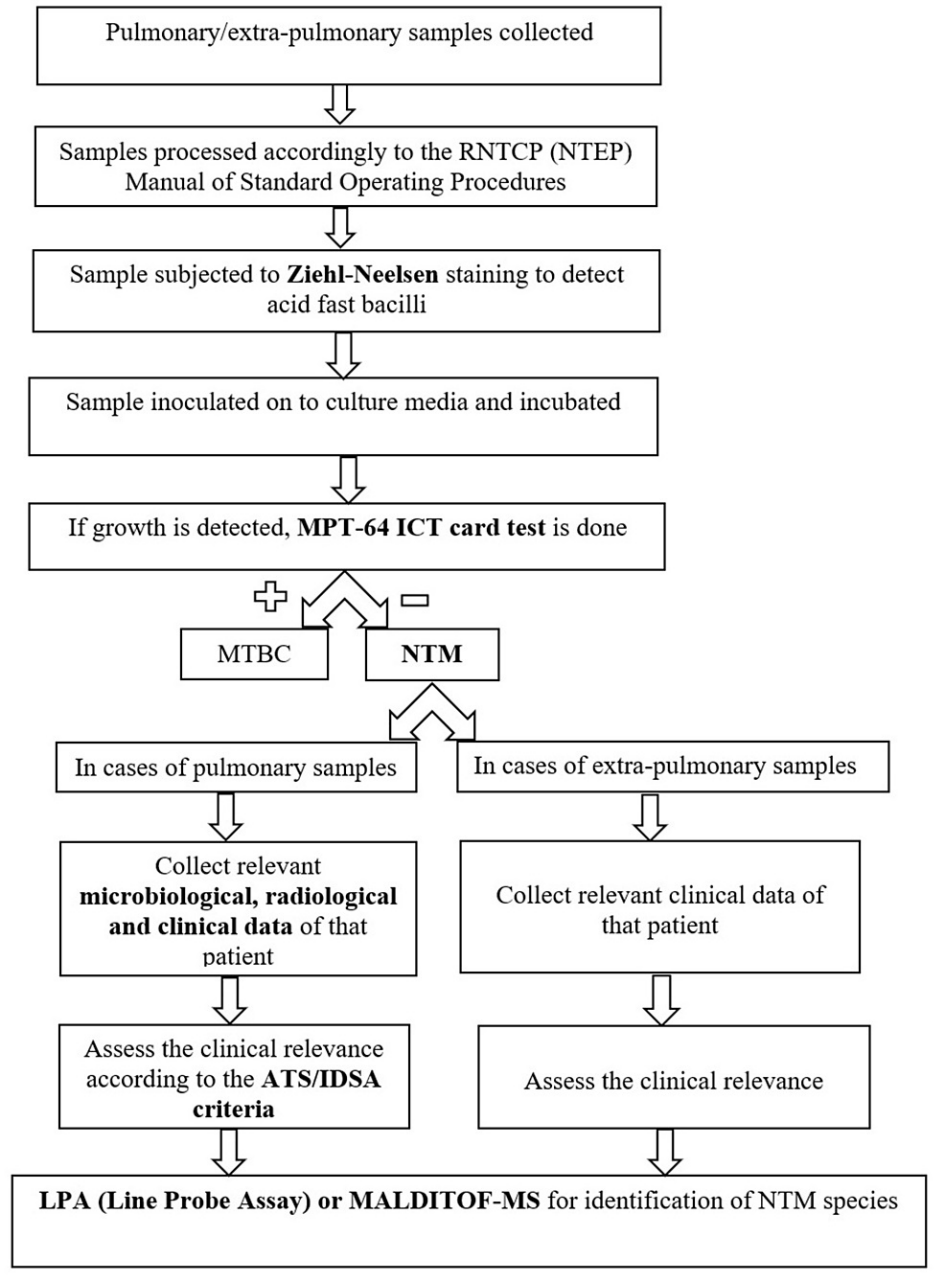
Speciation methodology used for identifying the species of non-tuberculous mycobacteria RNTCP (NTEP): Revised National Tuberculosis Control Programme (National Tuberculosis Elimination Programme); MPT-64 ICT: mpt64 antigen immunochromatographic test; MTBC: Mycobacterium tuberculosis; NTM: non-tuberculous mycobacteria; ATS/IDSA: American Thoracic Society and the Infectious Diseases Society of America; MALDI TOF MS: matrix-assisted laser desorption ionisation time of flight mass spectrometry; LPA: Line probe assay

Laparoscopic surgeries facilitate early patient discharge, often on the same day of surgery. Although it is greatly advantageous and improves the overall quality of care offered, it diminishes post-operative surveillance and increases the chances of missed PSIs [26]. Moreover, symptoms of delayed PSIs are non-specific and hence may be missed easily earlier in the course of the disease. Therefore, patients having undergone laparoscopic surgery must be carefully followed up and screened for signs of PSI. The diagnosis of NTM infections after laparoscopic surgeries typically involves a combination of imaging studies, microbiological culture, and histopathological examination of surgical specimens. Imaging modalities such as ultrasonography (USG) delineate the depth and the extent of the pathology. Higher-order imaging modalities such as contrast-enhanced computerised tomography (CT) and magnetic resonance imaging (MRI) may be deployed in patients with discharging sinuses to supplement the diagnosis. Following this, an extensive debridement ought to be carried out with excision of the lesion or the sinus tract. The usage of wound swabs for microbiological identification of the disease is fraught with inaccurate results, owing to the limited nature of the sample and the associated risk of desiccation [27]. Microbiological cultures of surgical specimens can confirm the presence of NTM which can later be speciated using MALDI TOF MS (matrix-assisted laser desorption ionisation time of flight mass spectrometry) or molecular techniques like LPA (line probe assay) (Figure 4) [28,29]. Histopathological examination can identify the presence of acid-fast bacilli in a milieu of chronic granulomatous inflammation and can also provide information about the extent of the infection.

There is a paucity of consensus regarding the treatment of delayed PSIs caused by NTM. Treatment typically involves a combination of antibiotics tailored to the specific NTM species and the patient's individual characteristics. NTM infections have a poor response to first-line anti-tubercular treatment [9]. However, treatment with second-line anti-tubercular drugs viz. macrolides, quinolones, tetracyclines, and aminoglycosides have shown promising results [9]. Table 4 summarises the treatment regimens reported by some recent studies. We devised a departmental protocol (Figure 1) for the management of port-site infections after reviewing existing literature and inviting opinions from surgeons, microbiologists, and infectious diseases experts. Patients of PSI presenting to our centre were managed according to the aforementioned departmental protocol. Patients with delayed PSIs were treated with a three-month course of oral clarithromycin and ciprofloxacin. Since NTM are elusive entities that frequently escape diagnosis, we also treated patients who had clinical features consistent with NTM infections even in the absence of microbiological or histopathological evidence of NTM. The patients responded well to treatment and no relapses were reported, highlighting the utility of second-line anti-tubercular agents in the treatment of NTM infections.

**Table 4:** A summary of recent studies describing the treatment regimen used to treat port-site infections. NTM: Non-tuberculous mycobacteria; PO: Per os (oral); IV: Intravenous

Serial	Study, NTM species	Treatment	Clinical Relapse
1.	Ghosh et al. [6], M. abscessus and M. fortuitum	Medical treatment based on drug sensitivity	No
2.	Ramesh et al. [14], M. tuberculosis	Surgical debridement + Standard first-line antitubercular regimen; rifampicin, isoniazid, pyrazinamide and ethambutol for 2 mo followed by rifampicin and isoniazid for 9 months	No
3.	Shah et al. [18], M. fortuitum, M. chelonae	Medical treatment only; clarithromycin (PO 6-9 months); ciprofloxacin (PO 6-9 months)	No
4.	Bhattacharjee et al. [19], 2015; species not specified	Medical treatment only; clarithromycin (PO 1-3 months); ciprofloxacin (PO 1-3 months)	Persistent skin nodules in 5 cases. Treated with amikacin (local injection 5 days)
5.	Chaudhuri et al. [5], 2010 M. fortuitum-chelonae complex	Medical treatment only; clarithromycin (PO 1-3 months) and ciprofloxacin (PO 1-3 months)	Persistent skin nodules in 7 cases. Treated with amikacin (local injection 5 days)
6.	Duarte et al. [20]; 2009, M. bolletii or M. massiliense	Surgical debridement + medical treatment; clarithromycin (PO 6 months); amikacin or imipenem or cefoxitin (IV 6 months)	No
7.	Vijayaraghavan et al. [21]; 2006; M. chelonae	Medical treatment only; ciprofloxacin (PO 2-18 months) and amikacin (IV 2-18 months)	No
8.	Krishnappa and Samarasam [22], 2017, M. chelonae and M. fortuitum	Surgical debridement and medical treatment; ciprofloxacin (PO 3 months) OR levofloxacin (PO 3 months) and clarithromycin (PO 3 months) and amikacin (IV 3 months)	Recurrence seen in three patients; medical retreatment with the same antibiotics
9.	Lahiri et al. [17], 2009, M. fortuitum	Surgical debridement + medical treatment; amikacin or imipenem (IV 6 weeks) and ciprofloxacin or clarithromycin (PO 6 weeks)	No
10.	Muthusami et al. [13], 2004, M. fortuitum	Surgical debridement + medical treatment; amikacin (IV 6 weeks-3 months) and ciprofloxacin (PO 6 weeks-3 months)	One patient relapsed after 3 months amikacin (IV; 3 months)
11.	Samaranayake et al. [23], 2008, NTM	Surgical excision of the sinus tract Medical treatment with trimethoprim-sulfamethoxazole (PO 3 months), ciprofloxacin (PO 3 months), and/or amikacin (IV 3 months)	No
12.	Wright et al. [25], 2014, M. fortuitum and M. abscessus	Medical treatment only amikacin, cefoxitin, and imipenem (IV 2-6 weeks); clarithromycin/doxycycline/ minocycline (PO 3-6 months); combination therapy with ciprofloxacin and cotrimoxazole used for M. fortuitum	All relapsed - required removal of the device (gastric band)
13.	Current study	Surgical excision of the sinus tract; medical treatment with clarithromycin (PO 3 months); ciprofloxacin (PO 3 months)	No relapse

On the flip side, however, there are reports of port-site abscesses presenting as a persistent discharging sinus months after surgery, which on investigation turned out to be due to a retained stone [30,31]. Thus, it is imperative to note that features consistent with delayed PSIs may arise even due to other factors. Clinicians, therefore, must resist the temptation to initiate antibiotic therapy before thoroughly working the patient up with appropriate imaging and microbiological investigations. Injudicious use of antibiotics must be avoided as it leads to the emergence of drug resistance.

The results reported herein should be considered in the light of some limitations. The first is the relatively small sample size of the study, which might have allowed certain confounding factors to remain unchecked. This restricts the generalisability of the findings detailed. Secondly, patient recruitment was carried out from the outpatient clinics. These patients had undergone surgery several weeks to months prior to presentation. Furthermore, the patients with delayed PSIs were more likely to present to the clinics, whereas patients with early PSIs would have benefited from empirical treatment, thereby obviating the need for a hospital visit. Therefore, the data pertaining to prior surgery and type of PSI were not granular. Thirdly, preoperative imaging was not carried out in a uniform manner, with patients being offered various imaging modalities. A more uniform approach to preoperative imaging might be preferred in subsequent studies. Lastly, the NTM identified were not speciated. Therefore, further studies with large sample sizes and a prospective study design may be conducted to provide better insight into delayed port-site infections.

## Conclusions

Port-site infections, although often seldom life-threatening, are a set of irksome complications that curtail the benefits of laparoscopic surgery. While early PSIs may be caused due to skin commensals and are fairly easy to treat, delayed PSIs caused by multidrug-resistant mycobacteria are treatment refractory. Robust sterilisation, with a departure from conventional glutaraldehyde-based techniques towards plasma and gas sterilisation, may reduce their incidence. Management of delayed PSIs involves a thorough microbiological work-up of the patients, followed by radical excision of the lesion with prolonged oral antibiotic therapy. Clinicians must not initiate empirical antibiotic therapy before investigating the patient as it may lead to the emergence of antimicrobial resistance.
